# Prediction of Cardiovascular Disease Risk by Cardiac Biomarkers in 2 United Kingdom Cohort Studies: Does Utility Depend on Risk Thresholds For Treatment?

**DOI:** 10.1161/HYPERTENSIONAHA.115.06501

**Published:** 2016-01-03

**Authors:** Paul Welsh, Carole Hart, Olia Papacosta, David Preiss, Alex McConnachie, Heather Murray, Sheena Ramsay, Mark Upton, Graham Watt, Peter Whincup, Goya Wannamethee, Naveed Sattar

**Affiliations:** From the Institute of Cardiovascular and Medical Sciences, BHF Glasgow Cardiovascular Research Centre (P.W., D.P., N.S.), Institute of Health and Wellbeing (C.H., G.W.), and Robertson Centre for Biostatistics (A.M., H.M.), University of Glasgow, Glasgow, United Kingdom; Department of Primary Care and Population Health, University College London, London, United Kingdom (O.P., S.R., P.W., G.W.); and Helmsley Medical Centre, Helmsley, York, United Kingdom (M.U.).

**Keywords:** adrenomedullin, biomarkers, cardiovascular diseases, natriuretic peptides, risk factors, troponin T

## Abstract

We tested the predictive ability of cardiac biomarkers N-terminal pro B-type natriuretic peptide (NT-proBNP), high-sensitivity troponin T, and midregional pro adrenomedullin for cardiovascular disease (CVD) events using the British Regional Heart Study (BRHS) of men aged 60 to 79 years, and the MIDSPAN Family Study (MFS) of men and women aged 30 to 59 years. They included 3757 and 2226 participants, respectively, and during median 13.0 and 17.3 years follow-up the primary CVD event rates were 16.6 and 5.3 per 1000 patient-years, respectively. In Cox models adjusted for basic classical risk factors, 1 SD increases in log-transformed NT-proBNP, high-sensitivity troponin T, and midregional pro adrenomedullin were generally associated with increased primary CVD risk in both the studies (*P*<0.006) except midregional pro adrenomedullin in MFS (*P*=0.10). In BRHS, QRISK2 risk factors yielded a C-index of 0.657, which was improved by 0.017 (*P*=0.005) by NT-proBNP, but not by other biomarkers. Using 28% 14-year risk as a proxy for 20% 10-year risk, NT-proBNP improved risk classification for primary CVD cases (case net reclassification index, 5.9%; 95% confidence interval, 2.8%–9.2%), but only improved classification of noncases at a 14% 14-year risk threshold (4.6%; 2.9%–6.3%). In MFS, ASSIGN risk factors yielded a C-index of 0.752 for primary CVD; none of the cardiac biomarkers improved the C-index. Improvements in risk classification were only seen using NT-proBNP and high-sensitivity troponin T among cases using the 28% 14-year risk threshold (4.7%; 1.0%–9.2% and 2.6%; 0.0%–5.8%, respectively). In conclusion, the improvement in treatment allocation gained by adding cardiac biomarkers to risk scores seems to depend on the risk threshold chosen for commencing preventative treatments.

## Abstract 2

Supplemental Digital Content is available in the text.

Stratified medicine for estimating cardiovascular disease (CVD) risk is a major responsibility of primary care.^1^ Health professionals use risk scores, such as ASSIGN, QRISK2, the Pooled Cohort Equations, and SCORE,^2–4^ to stratify and treat those at higher risk with statins, antihypertensive medications, and lifestyle advice as required. The American Heart Association/American College of Cardiology task force recently altered its definition of a high-risk treatment threshold in primary prevention from 20% 10-year risk to 7.5% 10-year risk.^5^ The National Institute of Health and Care Excellence in England and Wales also reduced the threshold, from 20% to 10% 10-year risk,^6^ whereas other national guidelines are still under revision.

Recently, cardiac biomarkers have become a major focus of attempts to improve CVD risk scores. Use of such biomarkers is attractive because they integrate signals from different pathophysiological pathways, including cardiac, vascular, and renal health. Data from several different cohort studies indicate that high-sensitivity troponins (hs-Tn) and N-terminal pro B-type natriuretic peptide (NT-proBNP)^7–10^ are strong predictors of CVD risk. A recent editorial from the Bypass Angioplasty Revascularization Investigation 2 Diabetes (BARI 2D) trial emphasises the importance of troponin data from cohort studies by suggesting that cardiac troponin values may become routinely used for risk stratification across the spectrum of ischemic heart disease.^11^ More recently, midregional pro adrenomedullin (MR-proADM) has also emerged as a biomarker of potential interest in CVD risk prediction.^12–14^ ADM, a natriuretic and diuretic peptide, is produced in human cardiac tissue (as well as adrenal glands, kidney tissues, and the vasculature) in response to mechanical stretch, much like natriuretic peptides. However, it is still not clear how much incremental information is gained by the use of multiple cardiac biomarkers in risk stratification, or whether they capture only overlapping risk information. Furthermore, although there is as yet no formal meta-analysis, the vast majority of cohort studies measuring cardiac biomarkers have focused on older and clinical trial cohorts, who will have substantially more prevalent and subclinical CVD than the young primary prevention groups CVD risk scores are intended for. Finally, in the context of national guidelines recommending lower thresholds of CVD risk for intervention with drugs to lower cholesterol or blood pressure, it is particularly important to test whether any refinement of CVD risk scores using cardiac biomarkers improves the specificity of risk prediction, because specificity falls as thresholds are lowered.^15^

Given these uncertainties, we aimed to investigate the ability of these 3 cardiac biomarkers to predict CVD in 2 United Kingdom cohort studies. The 20-year follow-up British Regional Heart Study (BRHS Q20) is a cohort of older British men, and the MIDSPAN Family Study (MFS) is a British cohort of younger men and women. The hypothesis was that cardiac biomarkers would improve clinical decision making in risk prediction models, but that changing treatment thresholds would alter their utility.

## Methods

### British Regional Heart Study

The BRHS is a socioeconomically representative prospective study involving 7735 men, aged 40 to 59 years, of predominantly white European ethnicity (>99%), drawn from 1 general practice in each of 24 British towns, who were screened between 1978 and 1980.^16^ In 1998 to 2000, all surviving men, then aged 60 to 79 years, were invited for a 20th year follow-up examination (Q20), on which the analyses presented here are based.^8^ Follow-up has been achieved for 99% of the cohort. Data relating NT-proBNP to CVD in BRHS have been previously published using a different modeling approach, and without other cardiac biomarkers.^8^

In BRHS, CVD events were defined as a composite of CVD death (all of those who died with *International Classification of Diseases Ninth Revision* 401 to 459 listed on the death certificate as a primary or secondary cause) and nonfatal myocardial infarction or stroke. Evidence of nonfatal myocardial infarction or stroke was obtained by ad hoc reports from general practitioners supplemented by biennial reviews of the patients’ practice records (including hospital and clinic correspondence) through to the end of the study period. A nonfatal myocardial infarction was diagnosed according to World Health Organisation criteria. Nonfatal stroke events were those that produced a neurological deficit that was present for >24 hours.^8^

### MIDSPAN Family Study

The MFS took place between March and December 1996. The study recruited adult sons and daughters of couples who had participated in the original Renfrew/Paisley prospective cohort study.^17^ In brief, offspring of the married couples identified within the Renfrew/Paisley cohort, aged 30 to 59 years and living locally, formed the eligible population (3202 offspring from 1767 families). In all, 1040 male and 1298 female offsprings from 1477 families took part, and all participants were white^18^.

End points were identified by periodic review of the cohort using a national database: the Information Services Division National Health Service record linkage for Scotland. The Information Services Division–linked database contains information on Scotland’s morbidity records for acute specialty day case and inpatient discharges from hospital (Scotland’s morbidity record 01) since January 1981. Death certificates were obtained from the National Health Service Central Register where the participants were flagged. For this study, the CVD end point was any event included in the national ASSIGN risk score definition of CVD: *International Classification of Diseases Tenth Revision* codes I20-25, G45, I60-69, as well as death from CVD (I00-I99), and OPCS4 procedure codes L29.5, L31.1, K40-46, K49, and K75 (procedures comprising carotid endarterectomy, carotid angioplasty, coronary artery bypass graft, and percutaneous transluminal coronary angioplasty).

### Biomarker Measurement

NT-proBNP and hsTnT were measured in plasma samples from both the studies on an automated clinically validated immunoassay analyzer (e411, Roche Diagnostics, Burgess Hill, United Kingdom) using the manufacturers’ calibrators and quality control reagents. MR-proADM was measured on an automated B.R.A.H.M.S Kryptor Compact plus (Thermo Fisher Scientific Hemel Hempstead, United Kingdom). The limit of detection was 5 pg/mL for NT-proBNP, 3 pg/mL for hsTnT, and 0.05 nmol/L for MR-proADM. Quality control materials >2 levels for each biomarker ran between 4.4% and 7.7% between runs.

### Statistics

From screening in 1998 to 2000 in the BRHS and 1996 in MFS, CVD events were based on follow-up to a first qualifying CVD event or censoring at a maximum 14.3 years of follow-up (median, 13.0 years) in BRHS and maximum 17.8 years of follow-up (median, 17.3 years) in MFS.

In both the studies, analyses were conducted for primary CVD events (defined as events occurring in the cohort after excluding those with previous CVD, either self-reported or occurring in previous surveys, and those taking statin medication at baseline) as well as all CVD events (ie, without the above exclusions). As a post hoc analysis, secondary CVD risk prediction was also tested in those with baseline CVD in the BRHS, but not in MFS because of lower power. All available data were used in all models, leading to models with more risk factors having fewer observations because of missing covariates.

Standard crude analyses and Cox proportional hazard models were used. C-indices were derived using the somersd package (STATA) used for survival data.^19^ C-indices were calculated in BRHS using predictors broadly based on those included in QRISK2 (the risk score used by National Institute of Health and Care Excellence for the United Kingdom^6^), and in MFS using predictors broadly based on those included in ASSIGN (the risk score used in Scotland^4^). Increased concordance was tested on addition of combinations of cardiac biomarkers. Improved prediction was also tested using the net reclassification index for survival data using the nricens package (R) with 5000 bootstraps.^20^ To improve comparability of the cohorts while maximizing study power for this metric, follow-up times for both the studies were censored at 14 years (representing the maximum available whole year of follow-up time of BRHS). The categorical net reclassification index was calculated using binary risk thresholds for clinical treatments of 14% 14-year risk and 28% 14-year risk (which were taken to ≈10% 10-year risk and 20% 10-year risk frequently cited in clinical guidelines).^6^ All analyses were performed in STATA (version 13.1) and R (version 3.1.1).

## Results

### Baseline Data

In BRHS, 3757 of 4252 male participants had complete baseline data for all 3 cardiac biomarkers (88.3%). At baseline across thirds of all 3 cardiac biomarkers, there was a trend for higher levels to be associated with higher risk demographic and cardiometabolic characteristics, with the exception that NT-proBNP and hsTnT were inversely associated with total cholesterol. A CVD event occurred in 788 participants, and the event rate was 21.0 per 1000 patient-years in the full cohort and 16.6 per 1000 patient-years in those without baseline CVD or statin prescription. Those who experienced an incident CVD event generally had more adverse classical CVD risk factor characteristics (see online-only Data Supplement).

In MFS, 2226 of 2338 participants had complete baseline data for all 3 cardiac biomarkers and consented to long-term follow-up (95.2%). Higher levels of NT-proBNP were associated with adverse risk factor characteristics (older age, chronic kidney disease, and higher baseline CVD prevalence) but also many protective characteristics (female sex, lower body mass index, enhanced lipid profile, and lower glucose/diabetes mellitus). In contrast, higher levels of hsTnT and MR-proADM were more consistently associated with adverse risk characteristics. In MFS, 195 experienced a CVD event, and the event rate was 5.8 per 1000 patient-years in the full cohort with biomarker measurements and 5.3 per 1000 patient-years in those without baseline CVD or statin prescription. Those who experienced an incident CVD event generally had a more adverse classical CVD risk factor characteristics (see online-only Data Supplement).

### Associations of Cardiac Biomarkers With CVD Risk

Across thirds of the biomarker distribution, elevated levels of all 3 cardiac biomarkers were associated with decreased event-free survival during the follow-up time (Figure). In BRHS, 1 SD increases in all 3 cardiac biomarkers because continuous variables were associated with increased risk of all CVD in extensively adjusted models (Table 1). Of the 3 cardiac biomarkers, NT-proBNP was the most strongly associated with risk. After cross adjusting for all 3 cardiac biomarkers (through inclusion in the same model; Table 1 model 3), both NT-proBNP and hsTnT remained associated with CVD risk, but the association of MR-proADM with all CVD outcomes was attenuated to the null. These results were consistent when the model was restricted to those without previous CVD or statin prescription, although the strength of the associations was somewhat attenuated to the null in all models.

**Table 1.**
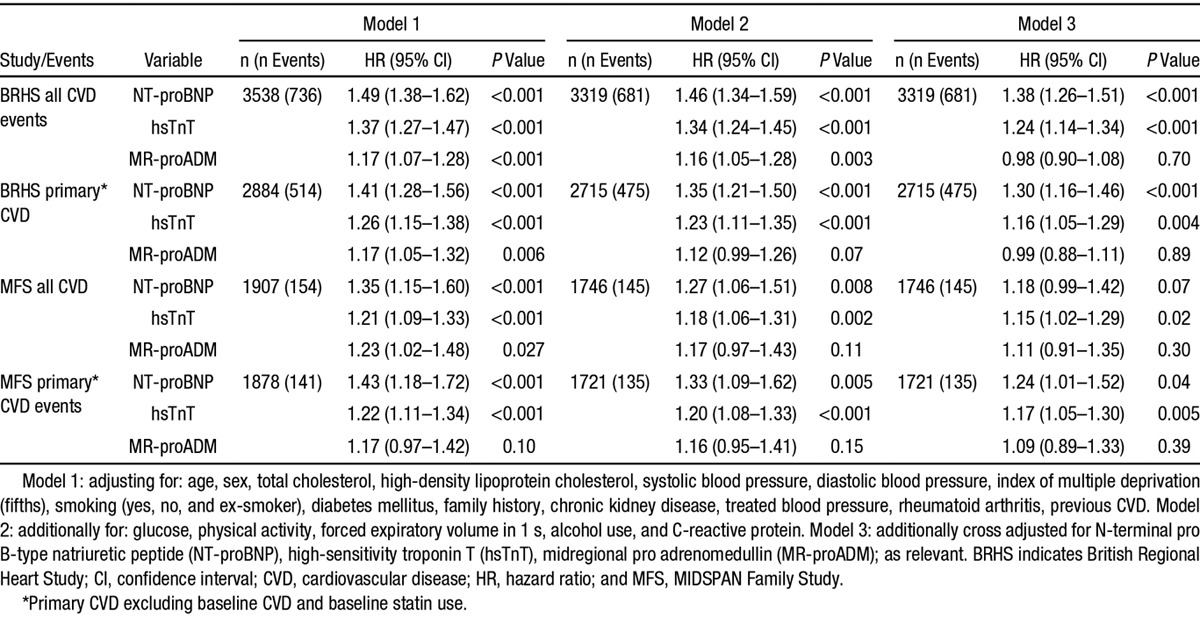
Associations of Cardiac Biomarkers (Per SD Increase on Log Scale) With CVD During Maximum Follow-Up Time

**Figure.**
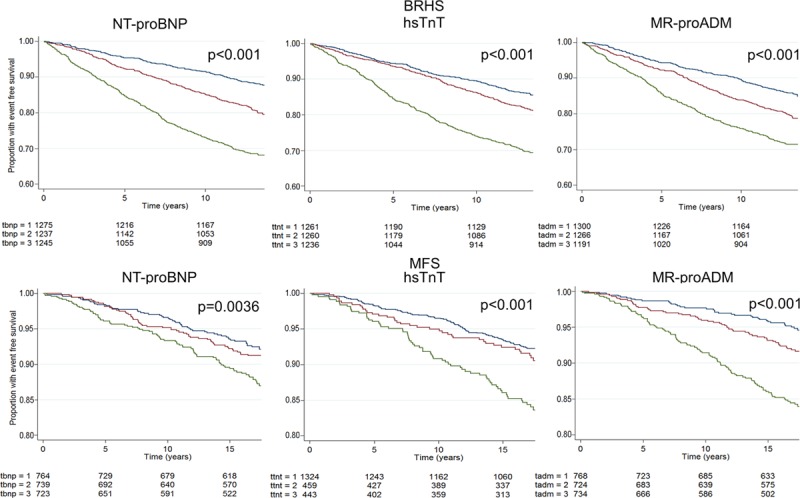
Kaplan–Meier curves showing cardiovascular disease event-free survival by thirds of all 3 cardiac biomarkers in both British Regional Heart Study (BRHS) and MIDSPAN Family Study (MFS). Blue line represents the lowest tertile (t1), red line intermediate (t2), and green top tertile (t3). Cut points ranges for thirds are defined in the online-only Data Supplement. *P* values are for log-rank tests. hsTnT indicates high-sensitivity troponin T; MR-proADM, midregional pro adrenomedullin; and NT-proBNP, N-terminal pro B-type natriuretic peptide.

In MFS, both NT-proBNP and hsTnT were positively associated with risk of all CVD and primary CVD (Table 1). NT-proBNP and hsTnT were more weakly associated with all CVD outcomes than in BRHS, but there was little difference in the strength of hazard ratios between the 2 cohorts for primary CVD, although confidence intervals were wider in MFS reflecting lower power. Cross adjustment for all 3 cardiac biomarkers in the same model attenuated results to the null, although both NT-proBNP and hsTnT retained a weak association with both all CVD and primary CVD (Table 1).

### Prediction of CVD in Risk Score Models

In BRHS participants without baseline CVD or previous statin prescription, a risk score based on factors included in QRISK2 yielded a C-index of 0.657 (Table 2). The c-index improved by 0.017 (*P*=0.005), 0.005 (*P*=0.28), and 0.005 (*P*=0.11) on addition of NT-proBNP, hsTnT, and MR-proADM, respectively. There was no evidence that combinations of biomarkers improved discrimination beyond the improvement gained from the addition of NT-proBNP. Data were essentially unchanged when ASSIGN score risk factors were used as the baseline predictor model in BRHS, therefore, demonstrating consistency of the results by different modeling approaches. In an exploratory post hoc analysis testing risk prediction of secondary CVD, hsTnT particularly strongly improved the discrimination of secondary CVD events (see online-only Data Supplement). Investigating risk category reclassification in primary CVD using binary models, NT-proBNP improved risk classification of 5.9% (95% confidence interval, 2.8%–9.2%) among cases (but not noncases) at a conservative 28% 14-year threshold. In contrast, NT-proBNP improved classification among noncases (4.6%; 2.9%–6.3%; but not cases) at a more radical 14% 14-year threshold (Table 3). Similar trends were observed for hsTnT. There was no evidence that MR-proADM improved risk classification in any model.

**Table 2.**
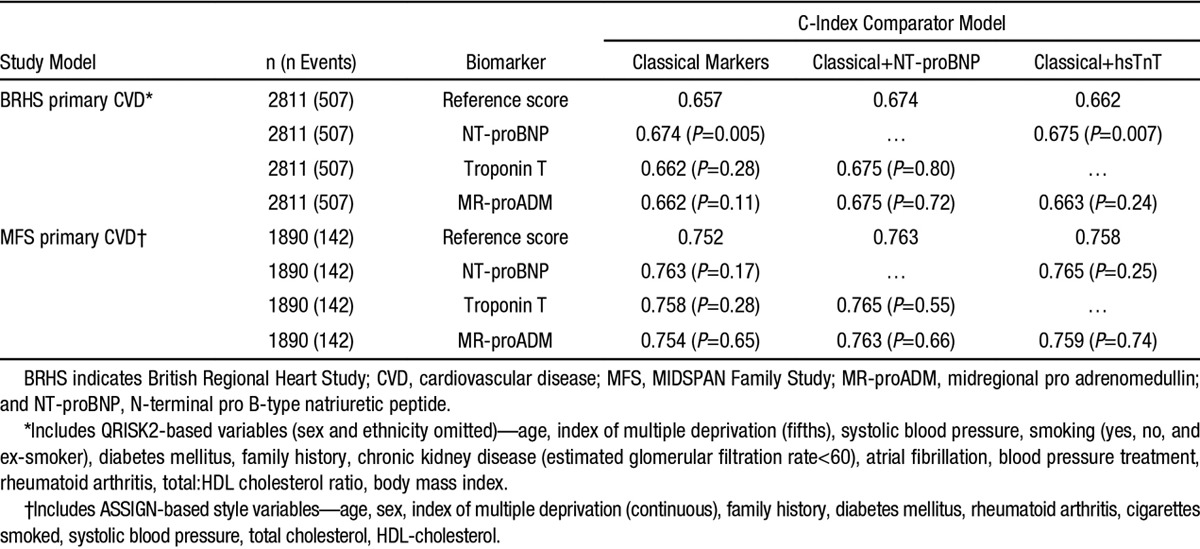
C-Index for the Prediction of Primary CVD (Among Those Not Taking Statin Medication at Baseline) by Cardiac Biomarkers in Addition to Risk Factors Based on Classical Risk Scores (QRISK2 and ASSIGN) During Maximum Follow-Up

**Table 3.**
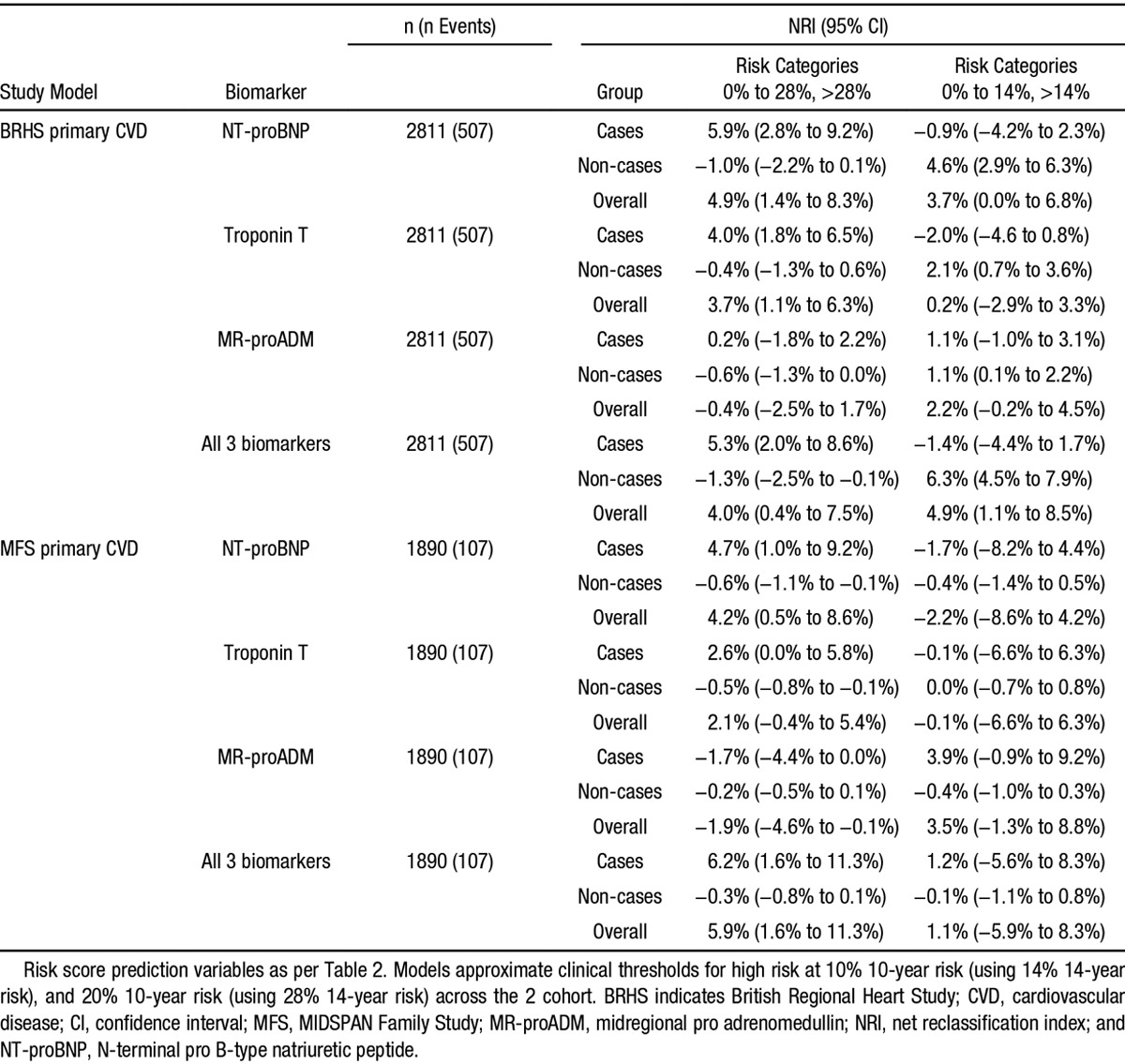
Cardiac Biomarker 14-Year NRIs for Primary CVD Prediction in Those Not Taking Statins at Baseline

In MFS participants without baseline CVD or previous statin prescription, a risk score based on risk factors included in ASSIGN (the risk score generally used in Scotland) yielded a C-index of 0.752 (Table 2). There was no evidence that individual or combined biomarkers improved discrimination. NT-proBNP improved risk classification only among cases at a 28% 14-year risk threshold by 4.7% (95% confidence interval, 1.0%–9.2%), but not at a 14% 14-year risk threshold. NT-proBNP did not improve the classification of noncases in any model (Table 3). hsTnT slightly improved risk classification only among cases at the 28% 14-year threshold. MR-proADM did not improve risk classification in any model.

## Discussion

In these 2 British cohort studies with a 23-year mean age difference and substantially different primary CVD event rates, the cardiac biomarker NT-proBNP only improved discrimination of CVD in the older BRHS cohort. Importantly, there was evidence that NT-proBNP and hsTnT improved classification of cases at a 28% 14-year risk threshold (theoretically resulting in more correct decisions to commence preventative treatment), but only improved classification of noncases at a 14% 14-year risk threshold (resulting in more correct decisions to not treat). As such, these biomarkers improved the sensitivity of risk prediction at the higher threshold, but improved specificity at the lower threshold. In contrast, MR-proADM was consistently a poor risk predictor of CVD. These data suggest that the clinical use (and health economics) of measuring these biomarkers in CVD risk stratification will depend on the risk threshold chosen for commencing preventative treatments, as well as characteristics of the screening population. These data are important to highlight in the context of ongoing changes to national guidelines for CVD risk scoring,^5,6^ particularly because the specificity of cardiovascular risk prediction falls as 10-year CVD risk thresholds are lowered.^15^

Recent mendelian randomization studies have shown that the active BNP hormone might protect against diabetes mellitus.^21^ Data from the Prospective Comparison of ARNi With ACE-I to Determine Impact on Global Mortality and Morbidity in Heart Failure (PARADIGM-HF) trial of the LCZ696 drug suggest that a neprilysin inhibitor, which prevents degradation of circulating natriuretic hormones is efficacious in improving outcomes and lowering blood pressure in the context of heart failure with reduced ejection fraction.^22^ As such, it is important to bear in mind that natriuretic peptides are physiologically protective hormones, and slightly elevated NT-proBNP in young people may not always be reflective of pathology. However, in older people with more comorbidity, elevated levels of natriuretic peptides become a more consistent biomarker of pathophysiological processes. In contrast to NT-proBNP, elevated troponin T seems to be a more consistent marker of characteristics that increase the risk of CVD in both the studies. Therefore characteristics of the risk screening population may have some bearing on how cardiac biomarkers perform as risk predictors.

The evidence of predictive ability of cardiac biomarkers in BRHS probably reflects (1) greater statistical power, (2) a greater burden of underlying subclinical disease in the older BRHS participants, and (3) a lower C-index in BRHS using classical risk factors compared with MFS. Cohorts that have hereto tested the use of cardiac biomarkers in CVD prediction have primarily comprised older participants at relatively high CVD risk, so the apparently low incremental discrimination gained from measuring cardiac biomarkers in the younger MFS is interesting. Indeed, our recent data from the Action in Diabetes and Vascular Disease: PreterAx and Diamicron MR Controlled Evaluation (ADVANCE) trial of patients with long-standing type 2 diabetes mellitus (a high-risk group) suggested that both NT-proBNP and hsTnT were much stronger predictors than was seen in the studies reported here.^10^ Recent data from Multi-Ethnic Study of Atherosclerosis (MESA) suggests that ethnicity is unlikely to importantly modify the predictive ability of cardiac biomarkers.^23^ Therefore, generalizability of cardiac biomarkers in CVD prediction is an ongoing area of interest. Large ongoing cohort studies, including Generation Scotland, as well as future meta-analyses will address these issues.

Strengths and weaknesses of the study require consideration. These are 2 large well-phenotyped prospective United Kingdom–based population studies, although small event numbers in MFS limited our power to observe small improvements in discrimination and risk classification. Differences between the studies include not only age but also geographic location, sex composition, and definitions of CVD. The differences in age at baseline allow a contrasting investigation of risk prediction, although BRHS only included male participants. Measurement of multiple cardiac biomarkers in the studies allowed assessment of the use of combinations of biomarkers. NT-proBNP and hsTnT are already routinely measured by automated methods in many routine biochemistry laboratories, and thus clinical translation potential is high. Risk prediction models are based on self-calibration in the cohorts, rather than using published risk scores. This was a decision made to prevent overestimation of the clinical use of the cardiac biomarkers. The improvements we see for discrimination using NT-proBNP are generally consistent with those seen for troponin I and BNP in a recent study in the Scottish Heart and Health Extended Cohort.^9^

## Perspectives

The ability of NT-proBNP and hsTnT to correctly influence clinical treatment decisions to prevent CVD was influenced by the risk threshold chosen for commencing preventative treatments, which is important in given recent changes to treatment thresholds in the guidelines. Meta-analysis and cost-effectiveness modeling are required to assess the use of cardiac biomarkers to aid risk prediction in a range of clinical settings.

## Acknowledgments

We thank Elaine Butler, Lynne Cherry, and Sara-Jane Duffus (University of Glasgow) for technical support.

## Sources of Funding

The British Regional Heart Study receives support from the British Heart Foundation Program grant (RG/08/013/25942). The MIDSPAN Family Study was funded by the Wellcome Trust and the National Health Service Cardiovascular Research and Development Program. None of the funding bodies were involved in the study design or the collection, analysis, and interpretation of data for this article or in the writing of the report. Dr Welsh is supported by British Heart Foundation fellowship FS/12/62/29889, which funded biomarker work.

## Disclosures

P. Welsh and N. Sattar hold a separate research grant from the Chief Scientist Office (Scottish Government Health and Social Care Directorates) relating to the use of cardiac biomarkers in cardiovascular disease risk prediction. The other authors report no conflicts.

## Supplementary Material

## Novelty and Significance

### What Is New?

- N-terminal pro B-type natriuretic peptide improved discrimination of future cardiovascular disease in the British Regional Heart Study (BRHS) cohort but not in the MIDSPAN Family Study cohort. In a model approximating the clinical 20% 10-year cardiovascular disease risk treatment threshold, inclusion of N-terminal pro B-type natriuretic peptide and high-sensitivity troponin T in risk scores improved treatment decisions among cases in both the studies. In contrast, in a model approximating the 10% 10-year treatment threshold, inclusion of cardiac biomarkers in the risk score in BRHS resulted in improved treatment decisions among noncases only.

### What Is Relevant?

- The ability of cardiac biomarkers to improve treatment decisions was influenced by the risk threshold chosen for commencing preventative treatments. This is important against a background where guidelines are changing treatment thresholds in clinical practice toward interventions being indicated at lower risk.

### Summary

Cardiac biomarkers still hold promise for cardiovascular disease risk prediction, but the changing landscape of the clinical risk prediction guidelines to identify high-risk patients may limit the ability of cardiac biomarkers to improve risk score sensitivity.
