# Supplementary material for: Prediction of Cardiovascular Disease Risk by Cardiac Biomarkers in 2 United Kingdom Cohort Studies: Does Utility Depend on Risk Thresholds For Treatment?
Source: Hypertension. 2016 Jan 3;67(2):309–15. doi: 10.1161/HYPERTENSIONAHA.115.06501 (PMC4716288; doi:10.1161/HYPERTENSIONAHA.115.06501)
Supplement: Supplementary file 1 [file hyp-67-309-s001.docx]

**ONLINE SUPPLEMENT**

**PREDICTION OF CARDIOVASCULAR DISEASE RISK BY CARDIAC BIOMARKERS IN TWO UK COHORT STUDIES: DOES UTILITY DEPEND ON RISK THRESHOLDS FOR TREATMENT?**

Paul Welsh^1^, Carole Hart^2^, Olia Papacosta^3^, David Preiss^1^, Alex McConnachie^4^, Heather Murray^4^, Sheena Ramsay^3^, Mark Upton^5^, Graham Watt^2^, Peter Whincup^3^, Goya Wannamethee^3^*, Naveed Sattar^1^*

*Joint senior authors

1 BHF Glasgow Cardiovascular Research Centre, University of Glasgow, Glasgow, UK

2 Institute of Health and Wellbeing, University of Glasgow, Glasgow, UK

3 Department of Primary Care and Population Health, University College London, London, UK

4 Robertson Centre for Biostatistics, University of Glasgow, Glasgow, UK

5 Helmsley Medical Centre, Helmsley, York, UK

**Short Title:** CARDIAC BIOMARKERS & CARDIOVASCULAR RISK PREDICTION

Correspondence to:

Paul Welsh, BHF Glasgow Cardiovascular Research Centre, 126 University Place, Glasgow G12 8TA

Tel: +441413302569 Fax: +441413306955

Email: paul.welsh@glasgow.ac.uk

**Supplementary methods**

**BRHS**

All men completed a mailed questionnaire providing information on their lifestyle and medical history, had a physical examination and provided a fasting blood sample. The samples were frozen and stored at -20^o^C on the day of collection and transferred for storage at -70^o^C until analysis. Baseline CVD, DM and rheumatoid arthritis, as well as family history of CVD, statin use, blood pressure medication use, and CKD were binary variables (1). Baseline CVD and diabetes was defined as either self-reported CVD (comprising MI or stroke)/diabetes or incident CVD/diabetes events occurring during follow-up in previous surveys. CKD was defined as an eGFR <60 ml/min/1.73 m2. Baseline RA was defined as taking any presumed rheumatoid arthritis treatment drug: BNF 10.1.2.1, 10.1.2.2, 10.1.3, and 10.1.5 although non-steroidal anti-inflammatory drugs (BNF 10.1.1) were not included due to indication for other conditions. Atrial fibrillation (AF) was diagnosed from ECG data at the study visit. Smoking was analysed as a categorical variable (never-smoker, ex-smoker, current-smoker). Alcohol use was from self-reported questionnaire data: never, occasional/light drinking (<1drink per week- 15 units of alcohol per week), or moderate/heavy classified as 16+ units per week or those who were unclassified. Physical activity from self-reported data: never, occasional/light, or moderate/vigorous. The Index of Multiple Deprivation (IMD) for England, Scotland and Wales was applied to the BRHS based on postcode of residence. IMD is a composite measure of deprivation available for small geographical areas (‘lower super output’ areas with an average of 1500 people). Fifths (quintiles) of IMD (qIMD) were defined according to national distribution of deprivation indices and applied to the BRHS. Family history of CVD was defined as self-report of mother or father dying of a “heart trouble” before the age of 60 (QRISK2 focuses on angina and MI before the age of 60, and not stroke).

All men provided written informed consent to the investigations, which were carried out in accordance with the Declaration of Helsinki, and approval was gained from relevant local ethics committees.

**MFS**

Where possible, covariate data was included in the same form as BRHS data (2–6). All information on physical activity, smoking, occupation, diet, socioeconomic status, and alcohol consumption were based on self-reported answers from standard questionnaires (5,6). Blood samples obtained were spun down, plasma separated, aliquoted, and stored at −80 C for subsequent analysis. Baseline CVD (MI/stroke) was defined as self-reported disease in the questionnaire. Baseline DM was defined as self-reported diabetes or fasting plasma glucose >7mmol/L. Baseline RA was self-reported and coded to ICD9 code 714.0. Smoking was analysed as a categorical variable (never-smoker, ex-smoker, current-smoker), but also was included as a continuous variable in some ASSIGN models according to number of cigarettes smoked per day. Alcohol use was from self-reported questionnaire data: never, occasional/light drinking (<1drink per week- 15 units of alcohol per week), or moderate/heavy classified as 16+ units per week or those who were unclassified. Physical activity from self-reported data of usual physical activities: never (“not at all physically active”), occasional/light (“not very physically active”), or moderate/vigorous (“fairly or very physically active”). The Scottish Index of Multiple Deprivation (SIMD) is a postcode based measure of deprivation developed specifically for Scotland, and used in the national CVD risk score (7). MFS participant deprivation was recorded using the earliest available (2004) SIMD data (8). Family history of CVD of CVD was defined as parental death from MI or stroke before the age of 60 or where discharge records show parents were admitted to hospital for CVD (ICD9 390-459) before the age of 60.

All participants provided written informed consent to the investigations, which were carried out in accordance with the Declaration of Helsinki, and approval was gained from local ethics committees and the Scottish Privacy Advisory Committee (PAC) for electronic record linkage.

**Statistical analysis**

Analyses were restricted to those with complete data for the three cardiac biomarkers at baseline, otherwise all available data were used. Subjects who went on to experience CVD events were compared to those who did not using medians and interquartile ranges (IQR) to summarize non-normally distributed data, means and standard deviation for normally distributed data, and frequencies and percentages for categorical data. Time-to-event curves were calculated for the three biomarkers across thirds of the distribution (using the lower limit of sensitivity as the bottom tertile for hsTnT) by the Kaplan–Meier method.

Normal distributions were approximated by taking logarithms of positively skewed cardiac biomarker variables for survival analyses. The associations of these markers with CVD risk were summarized using hazard ratios (HRs) derived from Cox proportional hazards models (proportional hazard assumptions were met in all cases), using continuous models (HR per 1 standard deviation [sd] increase) for circulating cardiac biomarkers. Adjustment models were fitted using the same markers in both studies for the Cox models as detailed in table legends; these generally had more risk factors than risk prediction models used clinically. In MFS overdispersion caused by familial clustering was tested using a model that allowed for frailty; this had no significant impact on data for any cardiac biomarker, and so models without a frailty component are presented.

QRISK 2 risk score variables include: Age, sex (all BRHS participants are male) , ethnicity (>99% of BRHS participant or of White European ethnicity), postcode based deprivation index, smoking status, diabetes status, angina or heart attack in a 1^st^ degree relative, CKD, AF, BP treatment, rheumatoid arthritis, total Chol/HDl ratio, SBP, BMI. (Available at <http://www.qrisk.org/>)

ASSIGN risk score variables include: Age, sex, postcode based deprivation index, Family history of CHD or stroke, diabetes status, rheumatoid arthritis, cigarettes smoked per day, SBP, total cholesterol, HDL cholesterol. (Available at <http://assign-score.com/estimate-the-risk/>)

**References**

1. Walker M. The British Regional Heart Study 1975-2004. *Int J Epidemiol*. 2004;33:1185–1192.

2. Hart CL, MacKinnon PL, Watt GCM, Upton MN, McConnachie A, Hole DJ, Davey Smith G, Gillis CR, Hawthorne VM. The Midspan studies. *Int J Epidemiol*. 2005;34:28–34.

3. Upton MN, McConnachie A, McSharry C, Hart CL, Smith GD, Gillis CR, Watt GC. Intergenerational 20 year trends in the prevalence of asthma and hay fever in adults: the Midspan family study surveys of parents and offspring. *BMJ.* 2000;321:88–92.

4. Welsh P, Doolin O, McConnachie A, Boulton E, McNeil G, Macdonald H, Hardcastle A, Hart C, Upton M, Watt G, Sattar N. Circulating 25OHD, dietary vitamin D, PTH, and calcium associations with incident cardiovascular disease and mortality: the MIDSPAN Family Study. *J Clin Endocrinol Metab.* 2012;97:4578–4587.

5. Hart CL, Davey Smith G, Upton MN, Watt GCM. Alcohol consumption behaviours and social mobility in men and women of the Midspan Family study. *Alcohol Alcohol.* 2009;44:332–336.

6. Abu-Rmeileh NME, Hart CL, McConnachie A, Upton MN, Lean MEJ, Watt GCM. Contribution of Midparental BMI and other determinants of obesity in adult offspring. *Obesity (Silver Spring)*. 2008;16:1388–1393.

7. Woodward M, Brindle P, Tunstall-Pedoe H. Adding social deprivation and family history to cardiovascular risk assessment: the ASSIGN score from the Scottish Heart Health Extended Cohort (SHHEC). *Heart.* 2007;93:172–176.

8. The Scottish Government. Scottish Index of Multiple Deprivation. Scottish Government. Available from: <http://www.gov.scot/Publications/2005/01/20458/49127>. Accessed November 11, 2015.

**Supplementary tables**

**S1** Characteristics across thirds of NT-proBNP in BRHS

**S2** Characteristics across thirds of hsTnT in BRHS

**S3** Characteristics across thirds of MR-proADM in BRHS

**S4** Characteristics across thirds of NT-proBNP in MFS

**S5** Characteristics across thirds of hsTnT in MFS

**S6** Characteristics across thirds of MR-proADM in MFS

**S7** Demographics, biochemistries and cardiac biomarkers by case status in both cohort studies

**S8** Sensitivity analysis using ASSIGN risk score variables in both cohorts. C-index for the prediction of primary CVD (in those not taking statin medication at baseline) by cardiac biomarkers in addition to risk factors based on ASSIGN over maximum follow-up. Data for MFS identical to table 3 in main paper (included for comparison to BRHS)

**S9** C-index for the prediction of secondary CVD using cardiac biomarkers in addition to risk factors based on classical risk scores (QRISK2 and ASSIGN) over maximum follow-up time

| **S1** Characteristics across thirds of NT-proBNP in BRHS | | | | |
| --- | --- | --- | --- | --- |
| Variable | T1 (n=1275)  ≤59pg/ml | T2 (n=1237)  60-142pg/ml | T3 (n=1245)  ≥143pg/ml | p-value |
| Age | 66.2 (4.6) | 68.7 (5.3) | 71.2 (5.4) | <0.001 |
| Smoking  -Never  -Ex  -Current | 428 (33.7%)  700 (55.0%)  145 (11.4%) | 346 (28.1%)  732 (59.4%)  155 (12.6%) | 317 (25.5%)  760 (61.1%)  167 (13.4%) | <0.001 |
| BMI | 26.9 (3.5) | 26.9 (3.6) | 26.8 (3.92) | 0.61 |
| IMD score | 19.6 (14.4) | 19.8 (14.4) | 21.4 (15.3) | 0.002 |
| SBP | 144.2 (20.6) | 149.2 (23.4) | 153.4 (26.9) | <0.001 |
| DBP | 85.1 (9.9) | 85.0 (10.9) | 85.0 (12.6) | 0.95 |
| TC | 6.19 (1.07) | 6.01 (1.05) | 5.80 (1.07) | <0.001 |
| HDL | 1.32 (0.33) | 1.32 (0.34) | 1.32 (0.36) | 0.97 |
| Glucose | 6.01 (1.95) | 5.99 (1.78) | 6.09 (2.00) | 0.37 |
| EGFR | 74.9 (11.1) | 73.3 (13.0) | 68.8 (13.5) | <0.001 |
| CKD (EGFR<60) | 110 (8.6%) | 162 (13.1%) | 295 (23.7%) | <0.001 |
| Physical activity  -Inactive  -Occasional/light  -Moderate/vigorous | 96 (7.8%)  488 (39.7%)  646 (52.5%) | 110 (9.2%)  498 (41.9%)  582 (48.9%) | 196 (16.4%)  540 (45.0%)  463 (38.6%) | <0.001 |
| Alcohol use  -None  -Occasional/light  -Moderate/heavy | 117 (9.3%)  897 (71.5%)  241 (19.2%) | 108 (8.9%)  864 (70.8%)  248 (20.3%) | 141 (11.5%)  842 (68.9%)  240 (19.6%) | 0.19 |
| Baseline CVD | 79 (6.3%) | 159 (13.1%) | 358 (29.3%) | <0.001 |
| Baseline DM | 85 (6.7%) | 78 (6.3%) | 110 (8.8%) | 0.031 |
| Family history | 62 (5.0%) | 60 (5.0%) | 86 (7.2%) | 0.028 |
| Statin use | 47 (3.7%) | 69 (5.6%) | 130 (10.4%) | <0.001 |
| BP med | 228 (18.1%) | 386 (31.6%) | 592 (48.3%) | <0.001 |
| CRP  (mg/L) | 1.24  (0.66, 2.71) | 1.56  (0.82, 3.39) | 2.07  (1.00, 4.55) | <0.001 |
| hsTnT  (pg/ml) | 10.1  (7.7, 13.5) | 11.4  (8.8, 15.3) | 14.6  (10.9, 19.8) | <0.001 |
| MR-proADM  (nmol/L) | 0.52  (0.46, 0.59) | 0.57  (0.49, 0.65) | 0.65  (0.56, 0.77) | <0.001 |
| Data are means (standard deviations), medians (interquartile ranges), or n (%)  BMI, body mass index; BP med, blood pressure medication; CKD, chronic kidney disease; CRP, C-reactive protein; CVD, cardiovascular disease; DBP, diastolic blood pressure; DM, diabetes mellitus eGFR, estimated glomerular filtration rate; HDL-c, high density lipoprotein cholesterol; IMD, index multiple deprivation; RA, rheumatoid arthritis; SBP, systolic blood pressure; TC, total cholesterol | | | | |

| **S2** Characteristics across thirds of hsTnT in BRHS | | | | |
| --- | --- | --- | --- | --- |
| Variable | T1 (n=1261)  ≤9.8pg/ml | T2 (n=1260)  9.9-14.2pg/ml | T3 (n=1236)  ≥14.3pg/ml | p-value |
| Age | 66.2 (4.6) | 68.9 (5.3) | 71.0 (5.5) | <0.001 |
| Smoking  -Never  -Ex  -Current | 415 (33.0%)  672 (53.5%)  170 (13.5%) | 363 (28.9%)  727 (57.8%)  168 (13.4%) | 313 (25.4%)  793 (64.2%)  129 (10.5%) | <0.001 |
| BMI | 26.4 (3.3) | 26.9 (3.6) | 27.3 (4.0) | <0.001 |
| IMD score | 19.3 (14.4) | 20.2 (14.6) | 21.3 (15.2) | 0.004 |
| SBP | 145.2 (22.6) | 148.9 (23.0) | 152.6 (25.9) | <0.001 |
| DBP | 85.0 (10.5) | 84.9 (10.9) | 85.3 (12.0) | 0.66 |
| TC | 6.06 (1.05) | 6.00 (1.07) | 5.95 (1.12) | 0.023 |
| HDL | 1.33 (0.34) | 1.32 (0.34) | 1.32 (0.35) | 0.55 |
| Glucose | 5.81 (1.24) | 5.99 (1.92) | 6.30 (2.38) | <0.001 |
| EGFR | 75.8 (11.6) | 72.4 (11.1) | 68.8 (14.5) | <0.001 |
| CKD (EGFR<60) | 100 (8.0%) | 166 (13.2%) | 301 (24.4%) | <0.001 |
| Physical activity  -Inactive  -Occasional/light  -Moderate/vigorous | 100 (8.2%)  522 (42.8%)  599 (49.1%) | 123 (10.2%)  480 (39.7%)  607 (50.2%) | 179 (15.1%)  524 (44.1%)  485 (40.8%) | <0.001 |
| Alcohol use  -None  -Occasional/light  -Moderate/heavy | 104 (8.3%)  886 (71.1%)  257 (20.6%) | 136 (11.0%)  881 (71.1%)  222 (17.9%) | 126 (10.4%)  836 (69.0%)  250 (20.6%) | 0.08 |
| Baseline CVD | 129 (10.4%) | 200 (16.2%) | 267 (22.1%) | <0.001 |
| Baseline DM | 59 (4.7%) | 81 (6.4%) | 133 (10.8%) | <0.001 |
| Family history | 70 (5.7%) | 60 (4.9%) | 78 (6.5%) | 0.23 |
| Statin use | 78 (6.2%) | 77 (6.1%) | 91 (7.4%) | 0.37 |
| BP med use | 321 (25.8%) | 396 (31.9%) | 489 (40.0%) | <0.001 |
| CRP  (mg/L) | 1.30  (0.70, 2.70) | 1.60  (0.84, 3.47) | 2.07  (0.99, 4.60) | <0.001 |
| NT-proBNP  (pg/ml) | 64  (35, 114) | 92  (49, 184) | 150  (70, 419) | <0.001 |
| MR-proADM  (nmol/L) | 0.53  (0.47, 0.61) | 0.57  (0.49, 0.65) | 0.63  (0.54, 0.77) | <0.001 |
| Data are means (standard deviations), medians (interquartile ranges), or n (%)  BMI, body mass index; BP med, blood pressure medication; CKD, chronic kidney disease; CRP, C-reactive protein; CVD, cardiovascular disease; DBP, diastolic blood pressure; DM, diabetes mellitus eGFR, estimated glomerular filtration rate; HDL-c, high density lipoprotein cholesterol; IMD, index multiple deprivation; RA, rheumatoid arthritis; SBP, systolic blood pressure; TC, total cholesterol | | | | |

| **S3** Characteristics across thirds of MR-proADM in BRHS | | | | |
| --- | --- | --- | --- | --- |
| Variable | T1 (n=1300)  ≤0.52nmol/L | T2 (n=1266)  0.53-0.62nmol/L | T3 (n=1191)  ≥0.63nmol/L | p-value |
| Age | 66.4 (4.8) | 68.6 (5.3) | 71.2 (5.4) | <0.001 |
| Smoking  -Never  -Ex  -Current | 491 (37.9%)  669 (51.6%)  136 (10.5%) | 342 (27.0%)  759 (60.0%)  164 (13.0%) | 258 (21.7%)  764 (64.3%)  167 (14.1%) | <0.001 |
| BMI | 25.9 (3.1) | 26.9 (3.4) | 27.9 (4.2) | <0.001 |
| IMD score | 18.7 (13.5) | 19.7 (14.7) | 22.6 (15.8) | <0.001 |
| SBP | 145.8 (21.9) | 149.9 (24.0) | 151.2 (25.9) | <0.001 |
| DBP | 84.9 (10.2) | 85.2 (11.1) | 85.0 (12.2) | 0.80 |
| TC | 6.02 (1.06) | 6.04 (1.06) | 5.95 (1.11) | 0.09 |
| HDL | 1.37 (0.35) | 1.31 (0.33) | 1.28 (0.34) | <0.001 |
| Glucose | 5.88 (1.53) | 6.03 (2.07) | 6.21 (2.09) | <0.001 |
| EGFR | 77.3 (11.5) | 73.8 (11.1) | 65.5 (12.9) | <0.001 |
| CKD (EGFR<60) | 72 (5.6%) | 111 (8.8%) | 384 (32.3%) | <0.001 |
| Physical activity  -Inactive  -Occasional/light  -Moderate/vigorous | 89 (7.1%)  479 (38.0%)  693 (55.0%) | 110 (9.0%)  514 (42.1%)  596 (48.9%) | 203 (17.9%)  533 (46.9%)  402 (35.3%) | <0.001 |
| Alcohol use  -None  -Occasional/light  -Moderate/heavy | 111 (8.7%)  950 (74.2%)  220 (17.2%) | 112 (9.0%)  884 (70.7%)  254 (20.3%) | 143 (12.3%)  769 (66.0%)  255 (21.9%) | <0.001 |
| Baseline CVD | 121 (9.5%) | 191 (15.4%) | 284 (24.4%) | <0.001 |
| Baseline DM | 79 (6.1%) | 80 (6.3%) | 114 (9.6%) | 0.001 |
| Family history | 64 (5.0%) | 71 (5.7%) | 73 (6.4%) | 0.36 |
| Statin use | 75 (5.8%) | 80 (6.3%) | 91 (7.6%) | 0.16 |
| BP med use | 254 (19.9%) | 388 (31.0%) | 564 (47.9%) | <0.001 |
| CRP  (mg/L) | 1.08  (0.57, 2.30) | 1.49  (0.85, 3.19) | 2.58  (1.30, 5.40) | <0.001 |
| hsTnT  (pg/ml) | 10.2  (7.9, 13.7) | 11.3  (8.7, 15.0) | 14.9  (10.8, 20.5) | <0.001 |
| NT-proBNP  (pg/ml) | 59  (31, 105) | 88  (49, 175) | 178  (86, 436) | <0.001 |
| Data are means (standard deviations), medians (interquartile ranges), or n (%)  BMI, body mass index; BP med, blood pressure medication; CKD, chronic kidney disease; CRP, C-reactive protein; CVD, cardiovascular disease; DBP, diastolic blood pressure; DM, diabetes mellitus eGFR, estimated glomerular filtration rate; HDL-c, high density lipoprotein cholesterol; IMD, index multiple deprivation; RA, rheumatoid arthritis; SBP, systolic blood pressure; TC, total cholesterol | | | | |

| **S4** Characteristics across thirds of NT-proBNP in MFS | | | | |
| --- | --- | --- | --- | --- |
| Variable | T1 (n=764)  ≤26pg/ml | T2 (n=739)  27-49pg/ml | T3 (n=723)  ≥50pg/ml | p-value |
| Age | 44.3 (5.7) | 45.4 (6.1) | 47.2 (6.2) | <0.001 |
| Sex male | 520 (68.1%) | 293 (39.7%) | 181 (25.0%) | <0.001 |
| Smoking  -Never  -Ex  -Current | 383 (50.1%)  157 (20.6%)  224 (29.3%) | 342 (46.3%)  194 (26.3%)  203 (27.5%) | 322 (44.5%)  207 (28.7%)  194 (26.8%) | 0.008 |
| BMI | 26.6 (4.1) | 25.8 (4.5) | 26.1 (5.1) | 0.001 |
| IMD score | 17.4 (13.6) | 18.9 (14.5) | 19.8 (14.7) | 0.005 |
| SBP | 127.8 (14.0) | 126.1 (15.6) | 127.1 (18.0) | 0.13 |
| DBP | 76.2 (10.6) | 74.3 (10.7) | 73.1 (12.3) | <0.001 |
| TC | 5.38 (0.93) | 5.18 (0.93) | 5.22 (1.01) | <0.001 |
| HDL | 1.34 (0.32) | 1.44 (0.37) | 1.47 (0.38) | <0.001 |
| Glucose | 5.58 (1.98) | 5.25 (1.33) | 5.23 (1.30) | <0.001 |
| eGFR | 103.0 (64.2) | 101.7 (25.5) | 99.6 (27.1) | 0.30 |
| CKD (EGFR<60) | 4 (0.5%) | 4 (0.6%) | 15 (2.1%) | 0.004 |
| Physical activity  -Inactive  -Occasional/light  -Moderate/vigorous | 65 (8.6%)  308 (40.4%)  390 (51.1%) | 39 (5.3%)  276 (37.4%)  424 (57.4%) | 50 (6.9%)  250 (34.6%)  423 (58.5%) | 0.01 |
| Alcohol use  -None  -Occasional/light  -Moderate/heavy | 111 (14.5%)  364 (47.6%)  289 (37.8%) | 128 (17.3%)  407 (55.1%)  204 (27.6%) | 148 (20.5%)  414 (57.3%)  161 (22.3%) | <0.001 |
| Baseline CVD | 3 (0.4%) | 3 (0.4%) | 19 (2.6%) | <0.001 |
| Baseline DM | 44 (5.8%) | 27 (3.7%) | 32 (4.4%) | 0.14 |
| Family history | 43 (5.7%) | 44 (6.0%) | 43 (6.0%) | 0.95 |
| Statin use | 5 (0.7%) | 3 (0.4%) | 6 (0.8%) | 0.59 |
| BP med use | 34 (4.5%) | 55 (7.4%) | 118 (16.3%) | <0.001 |
| CRP  (mg/L) | 0.78  (0.33-1.96) | 0.81  (0.34-1.98) | 1.06  (0.46-2.51) | <0.001 |
| hsTnT  (pg/ml) | 3.0  (3.0-4.0) | 3.0  (3.0-4.0) | 3.0  (3.0-4.4) | 0.15 |
| MR-proADM  (nmol/L) | 0.29  (0.23-0.35) | 0.29  (0.23-0.35) | 0.30  (0.24-0.39) | <0.001 |
| Data are means (standard deviations), medians (interquartile ranges), or n (%)  BMI, body mass index; BP med, blood pressure medication; CKD, chronic kidney disease; CRP, C-reactive protein; CVD, cardiovascular disease; DBP, diastolic blood pressure; DM, diabetes mellitus eGFR, estimated glomerular filtration rate; HDL-c, high density lipoprotein cholesterol; IMD, index multiple deprivation; RA, rheumatoid arthritis; SBP, systolic blood pressure; TC, total cholesterol | | | | |

| **S5** Characteristics across thirds of hsTnT in MFS | | | | |
| --- | --- | --- | --- | --- |
| Variable | T1 (n=1324)  <3pg/ml | T2 (n=459)  3.0-4.6pg/ml | T3 (n=443)  ≥4.7pg/ml | p-value |
| Age | 44.8 (6.0) | 46.1 (6.2) | 47.4 (6.2) | <0.001 |
| Sex male | 437 (33.0%) | 274 (59.7%) | 283 (63.9%) | <0.001 |
| Smoking  -Never  -Ex  -Current | 611 (46.2%)  389 (29.4%)  324 (24.5%) | 218 (47.5%)  96 (20.9%)  145 (31.6%) | 218 (49.2%)  73 (16.5%)  152 (34.3%) | <0.001 |
| BMI | 25.7 (4.3) | 26.6 (4.5) | 27.2 (5.0) | <0.001 |
| IMD score | 18.7 (14.4) | 18.0 (14.0) | 19.3 (14.4) | 0.40 |
| SBP | 124.8 (14.8) | 129.5 (16.0) | 131.2 (17.7) | <0.001 |
| DBP | 72.9 (10.5) | 76.4 (11.0) | 77.6 (12.6) | <0.001 |
| TC | 5.20 (0.94) | 5.31 (0.99) | 5.41 (0.97) | <0.001 |
| HDL | 1.44 (0.37) | 1.40 (0.38) | 1.37 (0.32) | 0.002 |
| Glucose | 5.26 (1.36) | 5.37 (1.42) | 5.62 (2.23) | <0.001 |
| eGFR | 102.9 (52.2) | 101.2 (25.8) | 97.4 (23.8) | 0.07 |
| CKD (EGFR<60) | 10 (0.8%) | 3 (0.7%) | 10 (2.3%) | 0.018 |
| Physical activity  -Inactive  -Occasional/light  -Moderate/vigorous | 86 (6.5%)  500 (37.8%)  738 (55.7%) | 42 (9.2%)  167 (36.5%)  249 (54.4%) | 26 (5.9%)  167 (37.7%)  250 (56.4%) | 0.31 |
| Alcohol use  -None  -Occasional/light  -Moderate/heavy | 230 (17.4%)  763 (57.6%)  331 (25.0%) | 81 (17.7%)  222 (48.4%)  156 (34.0%) | 76 (17.2%)  200 (45.2%)  167 (37.7%) | <0.001 |
| Baseline CVD | 13 (1.0%) | 3 (0.7%) | 9 (2.0%) | 0.11 |
| Baseline DM | 52 (4.0%) | 25 (5.5%) | 26 (5.9%) | 0.15 |
| Family history | 80 (6.0%) | 25 (5.5%) | 25 (5.6%) | 0.88 |
| Statin use | 6 (0.5%) | 3 (0.7%) | 5 (1.1%) | 0.30 |
| BP med use | 102 (7.7%) | 49 (10.7%) | 56 (12.7%) | 0.004 |
| CRP  (mg/L) | 0.84  (0.35-2.06) | 0.82  (0.38-1.90) | 1.06  (0.43-2.36) | 0.0074 |
| NT-proBNP  (pg/ml) | 35  (21-56) | 35  (21-54) | 40  (24-68) | 0.0027 |
| MR-proADM  (nmol/L) | 0.28  (0.22-0.34) | 0.32  (0.25-0.38) | 0.32  (0.26-0.40) | <0.001 |
| Data are means (standard deviations), medians (interquartile ranges), or n (%)  BMI, body mass index; BP med, blood pressure medication; CKD, chronic kidney disease; CRP, C-reactive protein; CVD, cardiovascular disease; DBP, diastolic blood pressure; DM, diabetes mellitus eGFR, estimated glomerular filtration rate; HDL-c, high density lipoprotein cholesterol; IMD, index multiple deprivation; RA, rheumatoid arthritis; SBP, systolic blood pressure; TC, total cholesterol | | | | |

| **S6** Characteristics across thirds of MR-proADM in MFS | | | | |
| --- | --- | --- | --- | --- |
| Variable | T1 (n=768)  ≤0.25nmol/L | T2 (n=724)  0.26-0.33nmol/L | T3 (n=734)  ≥0.34nmol/L | p-value |
| Age | 44.2 (5.8) | 45.4 (6.0) | 47.3 (6.2) | <0.001 |
| Sex male | 261 (34.0%) | 338 (46.7%) | 395 (53.8%) | <0.001 |
| Smoking  -Never  -Ex  -Current | 417 (54.3%)  142 (18.5%)  209 (27.2%) | 346 (47.8%)  184 (25.4%)  194 (26.8%) | 284 (38.7%)  232 (31.6%)  218 (29.7%) | <0.001 |
| BMI | 24.8 (3.6) | 26.0 (4.4) | 27.8 (5.1) | <0.001 |
| IMD score | 17.0 (13.3) | 18.0 (14.4) | 21.2 (14.9) | <0.001 |
| SBP | 123.6 (14.8) | 126.3 (15.6) | 131.3 (16.3) | <0.001 |
| DBP | 72.2 (10.7) | 74.3 (11.0) | 77.3 (11.5) | <0.001 |
| TC | 5.10 (0.93) | 5.27 (0.93) | 5.42 (1.00) | <0.001 |
| HDL | 1.47 (0.37) | 1.40 (0.35) | 1.37 (0.37) | <0.001 |
| Glucose | 5.18 (1.10) | 5.28 (1.50) | 5.61 (2.01) | <0.001 |
| EGFR | 104.1 (25.0) | 103.0 (67.2) | 97.1 (23.7) | 0.0037 |
| CKD (EGFR<60) | 5 (0.7%) | 5 (0.7%) | 13 (1.8%) | 0.054 |
| Physical activity  -Inactive  -Occasional/light  -Moderate/vigorous | 50 (6.5%)  267 (34.8%)  451 (58.7%) | 45 (6.2%)  287 (39.6%)  392 (54.1%) | 59 (8.1%)  280 (38.2%)  394 (53.8%) | 0.16 |
| Alcohol use  -None  -Occasional/light  -Moderate/heavy | 123 (16.0%)  477 (62.1%)  168 (21.9%) | 129 (17.8%)  377 (52.1%)  218 (30.1%) | 135 (18.4%)  331 (45.1%)  268 (36.5%) | <0.001 |
| Baseline CVD | 6 (0.8%) | 7 (1.0%) | 12 (1.6%) | 0.26 |
| Baseline DM | 21 (2.7%) | 25 (3.5%) | 57 (7.8%) | <0.001 |
| Family history | 49 (6.4%) | 43 (5.9%) | 38 (5.2%) | 0.60 |
| Statin use | 3 (0.4%) | 3 (0.4%) | 8 (1.1%) | 0.16 |
| BP med use | 42 (5.5%) | 61 (8.4%) | 104 (14.2%) | <0.001 |
| CRP  (mg/L) | 0.71  (0.29-1.69) | 0.75  (0.34-1.63) | 1.32  (0.58-3.03) | <0.001 |
| hsTnT  (pg/ml) | 3.0  (3.0-3.3) | 3.0  (3.0-4.0) | 3.1  (3.0-4.8) | <0.001 |
| NT-proBNP  (pg/ml) | 35  (21-54) | 34  (20-54) | 39  (24-67) | <0.001 |
| Data are means (standard deviations), medians (interquartile ranges), or n (%)  BMI, body mass index; BP med, blood pressure medication; CKD, chronic kidney disease; CRP, C-reactive protein; CVD, cardiovascular disease; DBP, diastolic blood pressure; DM, diabetes mellitus eGFR, estimated glomerular filtration rate; HDL-c, high density lipoprotein cholesterol; IMD, index multiple deprivation; RA, rheumatoid arthritis; SBP, systolic blood pressure; TC, total cholesterol | | | | |

| **S7** Demographics, biochemistries and cardiac biomarkers by case status in both cohort studies | | | | | | |
| --- | --- | --- | --- | --- | --- | --- |
| **Variable** | **BRHS** | | | **MFS** | | |
|  | No incident CVD (n=2969) | Incident CVD (n=788) | p-value | No incident CVD  (n=2031) | Incident CVD  (n=195) | p-value |
| Age (years) | 68.2 (5.4) | 70.5 (5.5) | <0.001 | 45.3 (6.1) | 49.0 (5.7) | <0.001 |
| Sex male | 3128 (100%) | 628 (100%) | - | 874 (43.0%) | 120 (61.5%) | <0.001 |
| Smoking  -Never  -Ex  -Current | 892 (30.1%)  1725 (58.2%)  349 (11.8%) | 199 (25.4%)  467 (59.6%)  118 (16.7%) | 0.006 | 980 (48.4%)  485 (23.9%)  564 (27.8%) | 65 (33.3%)  73 (37.4%)  57 (29.2%) | <0.001 |
| BMI (kg/m^2^) | 26.9 (3.7) | 27.0 (3.7) | 0.56 | 26.1 (4.5) | 26.9 (5.0) | 0.017 |
| IMD score* | 20.0 (14.6) | 21.4 (15.3) | 0.014 | 18.4 (14.1) | 22.2 (15.4) | <0.001 |
| SBP (mmHg) | 147.7 (23.3) | 153.3 (26.1) | <0.001 | 126.3 (15.5) | 134.6 (17.7) | <0.001 |
| DBP (mmHg) | 84.9 (10.9) | 85.6 (12.3) | 0.12 | 74.1 (11.1) | 79.2 (11.6) | <0.001 |
| Total-c (mmol/L) | 6.00 (1.08) | 6.00 (1.08) | 0.94 | 5.23 (0.95) | 5.59 (1.00) | <0.001 |
| HDL-c (mmol/L) | 1.33 (0.34) | 1.28 (0.33) | <0.001 | 1.43 (0.36) | 1.30 (0.34) | <0.001 |
| Glucose (mmol/L) | 5.95 (1.70) | 6.34 (2.53) | <0.001 | 5.30 (1.37) | 5.97 (2.95) | <0.001 |
| eGFR | 73.1 (12.6) | 70.0 (13.4) | <0.001 | 101.6 (44.7) | 100.4 (23.5) | 0.71 |
| CKD (eGFR<60) | 404 (13.6%) | 163 (20.7%) | <0.001 | 22 (1.1%) | 1 (0.5%) | 0.45 |
| Physical activity  -Inactive  -Occasional/light  -Moderate/vigorous | 281 (9.8%)  1201 (41.8%)  1389 (48.4%) | 121 (16.2%)  325 (43.5%)  302 (40.4%) | <0.001 | 134 (6.6%)  775 (38.2%)  1121 (55.2%) | 20 (10.3%)  59 (30.3%)  116 (59.5%) | 0.03 |
| Alcohol use  -None  -Occasional/light  -Moderate/heavy | 273 (9.3%)  2073 (70.8%)  581 (19.9%) | 93 (12.1%)  530 (68.7%)  148 (19.2%) | 0.08 | 345 (17.0%)  1100 (54.2%)  586 (28.9%) | 42 (21.5%)  85 (43.6%)  68 (34.9%) | 0.014 |
| Baseline CVD | 378 (13.0%) | 218 (28.0%) | <0.001 | 10 (0.5%) | 15 (7.7%) | <0.001 |
| Baseline DM | 187 (6.3%) | 86 (10.9%) | <0.001 | 83 (4.1%) | 20 (10.4%) | <0.001 |
| Baseline RA | 7 (0.2%) | 3 (0.4%) | 0.48 | 6 (0.3%) | 1 (0.5%) | 0.61 |
| Family history CVD | 150 (5.5%) | 48 (6.3%) | 0.41 | 115 (5.7%) | 15 (7.7%) | 0.25 |
| Statin use | 172 (5.8%) | 74 (9.4%) | <0.001 | 10 (0.5%) | 4 (2.1%) | 0.009 |
| BP med use | 867 (29.6%) | 339 (43.6%) | <0.001 | 167 (8.2%) | 40 (20.5%) | <0.001 |
| CRP  (mg/L) | 1.50  (0.78, 3.27) | 2.00  (1.00, 4.35) | <0.001 | 0.84  (0.36-2.00) | 1.34  (0.60-3.04) | <0.001 |
| NT-proBNP  (pg/ml) | 82  (43, 169) | 148  (72, 392) | <0.001 | 35  (21-56) | 41  (23-73) | 0.002 |
| hsTnT  (pg/ml) | 11.3  (8.6, 15.4) | 14.1  (10.2, 19.6) | <0.001 | 3.0  (3.0-4.0) | 3.1  (3.0-5.5) | <0.001 |
| MR-proADM (nmol/L) | 0.56  (0.49, 0.65) | 0.61  (0.53, 0.73) | <0.001 | 0.29  (0.23-0.35) | 0.34  (0.28-0.42) | <0.001 |
| Data are means (standard deviations), medians (interquartile ranges), or n (%)  BMI, body mass index; BP med, blood pressure medication; CKD, chronic kidney disease; CRP, C-reactive protein; CVD, cardiovascular disease; DBP, diastolic blood pressure; DM, diabetes mellitus eGFR, estimated glomerular filtration rate; HDL-c, high density lipoprotein cholesterol; IMD, index multiple deprivation; RA, rheumatoid arthritis; SBP, systolic blood pressure; TC, total cholesterol  * Higher score indicates more deprived | | | | | | |

| **S8** Sensitivity analysis using ASSIGN risk score variables in both cohorts. C-index for the prediction of primary CVD (in those not taking statin medication at baseline) by cardiac biomarkers in addition to risk factors based on ASSIGN over maximum follow-up. Data for MFS identical to table 3 in main paper (included for comparison to BRHS). | | | | | |
| --- | --- | --- | --- | --- | --- |
| **Study model** | **N (n events)** | **Biomarker** | **C-index comparator model** | | |
|  |  |  | **Classical markers** | **Classical + NT-proBNP** | **Classical + hsTnT** |
| **BRHS primary CVD** | 2936 (523) | Reference score | 0.650 | 0.667 | 0.654 |
|  | 2936 (523) | NT-proBNP | 0.667  (p=0.007) | - | 0.667  (p=0.01) |
|  | 2936 (523) | Troponin T | 0.654  (p=0.33) | 0.667  (p=0.97) | - |
|  | 2936 (523) | MR-proADM | 0.655  (p=0.06) | 0.667  (p=0.72) | 0.655  (p=0.29) |
|  |  |  |  |  |  |
| **MFS primary CVD** | 1890 (142) | Reference score | 0.752 | 0.763 | 0.758 |
|  | 1890 (142) | NT-proBNP | 0.763  (p=0.17) | - | 0.765  (p=0.25) |
|  | 1890 (142) | Troponin T | 0.758  (p=0.28) | 0.765  (p=0.55) | - |
|  | 1890 (142) | MR-proADM | 0.754  (p=0.65) | 0.763  (p=0.66) | 0.759  (p=0.74) |
| Classical risk factors include ASSIGN-based style variables – age, sex, IMD (continuous), family history, diabetes, RA, cigs smoked, SBP, total cholesterol, HDL-cholesterol | | | | | |

| **S9** C-index for the prediction of secondary CVD using cardiac biomarkers in addition to risk factors based on classical risk scores (QRISK2 and ASSIGN) over maximum follow-up time | | | | | |
| --- | --- | --- | --- | --- | --- |
| **Study model** | **N (n events)** | **Biomarker** | **C-index comparator model** | | |
|  |  |  | **Classical markers** | **Classical + NT-proBNP** | **Classical + hsTnT** |
| **BRHS secondary CVD*** | 554 (204) | Reference score | 0.613 | 0.639 | 0.660 |
|  | 554 (204) | NT-proBNP | 0.639  (p=0.036) | - | 0.665  (p=0.17) |
|  | 554 (204) | Troponin T | 0.660  (p=0.008) | 0.665  (p=0.079) | - |
|  | 554 (204) | MR-proADM | 0.612  (p=0.82) | 0.640  (p=0.59) | 0.661  (p=0.48) |
| * Includes QRISK 2-based variables (sex and ethnicity omitted) – age, IMD (fifths), SBP, smoking (yes, no, ex), diabetes, family history, CKD (EGFR<60), AF, BP treatment, RA, total:HDL cholesterol ratio, BMI. | | | | | |
